# Drowning is fast, silent, and preventable: a Texas example of research in action

**DOI:** 10.1186/s40621-023-00480-3

**Published:** 2023-12-12

**Authors:** Stewart R. Williams, Emily A. Dow, Molly B. Johnson

**Affiliations:** 1 Trauma Services, Dell Children’s Medical Center, Austin, TX USA; 2 Kinesiology Department, University of the Incarnate Word, San Antonio, TX USA; 3 Trauma and Injury Research Center, Dell Children’s Medical Center, Austin, TX USA

**Keywords:** Swimming, Drowning, Submersion, Texas, Injury prevention, Editorial

## Abstract

Drowning is a major public health issue internationally. In August 2022, a report was released by members of the Central Texas Drowning Prevention Action Team that provided data on drowning fatalities in Texas between 2006 and 2020 and offered recommendations for drowning prevention actions. The information in the Texas drowning report is an important contribution to the field of injury prevention. The aim of this editorial is to allow the information in the report to be available to a wider audience and potentially used as a model for other states.

## Editorial

A new report from Texas can increase the awareness of drowning as a public health issue by combining data with community education and outreach to strengthen drowning prevention efforts. The August 2022 report, *One Texan dies from drowning every day! A report on fatal unintentional drownings, Texas, 2006–2020* presented data on fatal drownings by Texans over a 15-year period [1]. The report was authored by members of the Central Texas Drowning Prevention Action Team, which brings together swim teachers, first responders, policy experts, and other drowning prevention stakeholders, such as those from state agencies. The report was a collaborative effort with Colin’s Hope, an Austin-based non-profit highly active in drowning prevention, and the Dell Children’s Medical Center, which established a Drowning Prevention and Water Safety Program in 2020 with a research scientist and an injury prevention specialist devoted to drowning prevention. This work presented important content in an accessible format for non-researchers and is an example of the power of partnership between research and injury prevention practice in the community.

Using free, publicly available data from CDC WONDER, the report found that 5401 Texans died due to unintentional drowning during the 15-year time period between 2006 and 2020. There were more fatal drownings and a higher death rate due to drowning in 2020 than all 14 prior years. At-risk populations mirrored what is found across the US: drowning death rates were highest for males, Black residents, and children 1–4 years old. More 1–4 years old died from drowning than from any other cause. Although natural water was the setting with the most fatal drownings overall, younger age groups were more likely to drown in other water settings. Bathtubs posed the highest risk for infants under 1 year old. Swimming pools posed the highest risk for children 1–4 and 5–14 years old. Natural water posed the highest risk for adults and teenagers 15 years and older.

The report offered detailed suggestions for outreach, research, and policy at all points in the drowning timeline: during preparation, prevention, reaction, and mitigation phases. Recommendations for preparation highlighted commencing water safety education in the prenatal period and continuing through childhood and adulthood, improving caregiver preparation prior to water-based activities by children (such as gathering towels and clothes before bathing an infant), and posting CPR instructions at aquatic venues. Suggestions to make water time safer included designating a water watcher (whose sole responsibility is to supervise children around water) and providing careful supervision of infants and children during transition points (such as eating or packing to leave). Suggestions to improve responsiveness advised caregivers to search for missing children by first checking for the possibility of drowning, to include rescue breathing during resuscitation of a drowning victim, and to consider the water competency of the caregiver when making decisions about supervision and rescue. Suggestions for mitigation highlighted the need to support families impacted by drowning. Future work could address the current debate about whether constant flotation device use by young children can provide a false sense of security that may put them at risk when they are not wearing any flotation devices.

### Water safety education

This report supported the need to tailor water safety education to groups at increased risk, such as toddlers and males, and suggests the importance of developing water competency for both children and adults. The report indicated that Black Texans had a higher death rate compared to people of other races. It is essential we understand what factors contribute to racial disparities in drowning rates, swimming capability, and swim lesson accessibility so we can work to reduce barriers for higher risk populations.

The report highlighted gaps in current approaches to water safety education. For example, the water setting with the highest number of drownings in Texas was natural water, yet most swimming lessons are taught in swimming pools. There may be a need to address natural water hazards in swimming lessons and potentially to offer swimming lessons in natural water. In addition to the suggestions in the report, we recommend finding ways to incorporate water safety into school curriculum. Schools could implement a water safety week just as there is a Fire Safety Week and could integrate water safety and competency into traditional academic courses. Additionally, we support recommendations that water safety education include discussion of protective measures beyond water competency, such as using life jackets, avoiding drugs and alcohol when boating, and knowing how to identify and respond to hazards (such as rip currents and bad weather).

### Injury prevention

This report added value to injury prevention by describing the magnitude of fatal drownings in Texas. Drowning prevention should be a priority so that our communities can be made more aware of how critical the issue is, interventions can be developed to reduce drowning, and policies can be enacted to ensure people remain safe around all types of water.

While education is important, injury prevention activities should include other layers as well. Injury prevention specialists should review local drowning data to understand additional community risks associated with drowning that can be targeted for intervention. Examples of avenues we suggest exploring include the following: (1) whether different municipal pool fencing regulations impact residential pool drowning rates; (2) if there are geographic patterns in the community related to bathtub drownings; (3) if it is possible to map open-water sites with high levels of recreational use and/or drowning occurrence; or (4) if drowning prevention devices or signage are available at these sites to assist first responders. Often, there are many opportunities to impact drowning occurrences such as signage, device placement, or policy without having to engage in resource intensive education efforts. Additionally, future reports should include county-level data, which is available in CDC WONDER and could support local/regional injury prevention efforts.

### Research

This report highlighted gaps in knowledge that needs to be addressed with further research. The report showed higher drowning rates for Black Texans compared with other races for both adults and children. In the future work, analyzing data over a longer time period may yield greater insights into potential disparities among specific age groups.

With only fatal, unintentional drownings included in the report, information on non-fatal drownings is needed. Since minor incidents may not involve medical intervention and therefore tracking. Research is needed that combines data from many sources to better understand the full scope of fatal and non-fatal drowning. Additionally, it will be important to fill out details about the circumstances surrounding drowning incidents to expand information about supervision in children’s submersions, to highlight the role of alcohol or drugs on those who drown or their supervisors, and to explore the role of swimming competency on drowning in all age groups. Recommendations for protective actions provided in the report could, then, be substantiated by data about drowning circumstances.

This report used CDC WONDER data to overview drowning fatalities in Texas. Due to the accessibility of the data, it bypasses the potential challenges of accessing and linking data from medical examiners and child fatality review teams across Texas. However, CDC WONDER data are limited because it does not include recent drowning trends. Future work should address the need for surveillance of drowning injuries in real time.

## Conclusion

The new drowning report from Texas is a novel example of one state’s approach to use data to guide drowning prevention and water safety education efforts, research, and policy development. It can be used as a model to improve data-driven drowning prevention efforts in different states and regions.

## Data Availability

Not applicable.

## Abbreviations

Black: Black or African American
CPR: Cardiopulmonary resuscitation
CDC: Centers for Disease Control and Prevention
WONDER: Wide-Ranging Online Data for Epidemiologic Research
